# Do China rural traditional Chinese medicine hospitals provide efficient healthcare to the people? Empirical study from 2013 to 2018 using data envelopment analysis

**DOI:** 10.1371/journal.pone.0267490

**Published:** 2022-04-22

**Authors:** Qian Bai, Xiaowei Man, Baolin Hong, Bo Li, Xuefeng Shi, Ying Bian

**Affiliations:** 1 State Key Laboratory of Quality Research in Chinese Medicine, Institute of Chinese Medical Sciences, University of Macau, Taipa, Macau, China; 2 School of Management, Beijing University of Chinese Medicine, Beijing, China; 3 Jinan Municipal Hospital of Traditional Chinese Medicine, Jinan, China; Northeastern University (Shenyang China), CHINA

## Abstract

Rural traditional Chinese medicine hospitals bear responsibilities of providing efficient medical services for rural residents. Efficiency assessments have previously been conducted in single province. This study aimed to investigate the technical efficiency of rural traditional Chinese medicine hospitals across China from 2013 to 2018, with the application of super slack-based measure data envelopment analysis. In total, 1219 hospitals covering 28 provinces were included as sample hospitals. Overall, hospitals performed technically less efficiently but presented with an increasing trend. Redundancy and insufficiency existed in health input and output variables, respectively. Notably, optimizing input variables was found to make more substantial improvement in hospital efficiency. Provincial and regional disparities were also observed in hospital efficiency. In conclusion, rural traditional Chinese medicine hospitals have experienced slight improvement in efficiency during the study period, however, their efficiency was still in a relatively low level with ample room for improvement. Meanwhile, regional coordinated development should also be noticed in this process.

## Introduction

Optimizing hospital efficiency is proven to be an affordable and necessary approach to accommodate medical needs of the public, given the scarcity of healthcare resources. In America, by enhancing hospital efficiency, bed occupancy rate is expected to rise by at least 15% without adding extra beds or staff [1]. China also faces a severe scarcity of healthcare resources, and the government has always made efforts to provide accessible medical services for the nation. However, many people continue to lack access to rational healthcare, particularly in rural areas [2, 3]. Around 2.4% of rural patients do not receive any treatment within two weeks, which is apparently higher than 0.7% in urban patients [4]. As the hub of rural three-level health care delivery system, rural hospitals are the major providers for rural residents. It was estimated that rural hospitals, which accounted for 47.03% in amount, offered merely 33.63% outpatient and emergency services, as well as 46.45% inpatient services [5]. Rural hospitals have not made full use of existing healthcare resources to serve surrounding locals. Government initiated healthcare reform targeting public rural hospitals in 2012, and it released another official document to promote reform progress of public rural TCM hospitals in 2015. This round of reforms attempts to enhance rural hospital efficiency by improving compensation and inner management system and eventually establishing a universally covered healthcare system.

Hospital efficiency evaluation has long been a hotpot in academia. Data envelopment analysis (DEA) is frequently applied since it does not rely on a specific function form and is suitable for multiple inputs and outputs [6, 7]. Gulnur Ilgun [8], Yusefzadeh [9], Polyzos [10], Mujasi [11], Tom Achoki [12], and Li L [13] constructed traditional CCR (named after Charnes, Cooper, Rhodes) and BCC (named after Banker, Charnes, Cooper) DEA models for measuring hospital efficiency in Turkey, Iran, Greece, Uganda, Zambia, and China. Gradually, scholars spotted some pitfalls of conventional DEA models and derived DEA models emerged. Tone [14] pioneered a slack-based measure (SBM) DEA model in 2001, which directly calculated the input excess and output insufficiency of evaluated units and circumvented the shortage of not considering slacks in traditional models. Additionally, he proposed super-SBM DEA model, aiming to distinguish the best performers evaluated by SBM DEA model [15]. Liu Q [16] and Liu WL [17] evaluated community health services and rural health expenditure efficiency in China using super-SBM DEA model.

As a unique medical system in China, traditional Chinese medicine (TCM) can diagnose and treat various illnesses using easy-to-operate techniques, such as acupuncture, cupping, and moxibustion. Moreover, TCM services were found to be 2–30 times less expensive than modern medicine services while exerting similar therapeutic effect towards certain diseases [18]. Therefore, TCM is deemed to be particularly crucial for rural areas which is more susceptible to health resource and economical constraints [19]. As the dominating supplier of grassroots TCM, how efficiently rural TCM hospitals operate will exert vital impacts on the health condition of residents. However, there was an alarmingly large degree of inefficiency in rural TCM hospitals. Zhang C and Wang XJ [20] found the average efficiency of rural TCM hospitals was merely 0.684 in Heilongjiang province, even only 15.8% of sample hospitals were technically efficient. Similarly, Sun X et al. [21] revealed inefficiency existed in nearly 80% of sample rural TCM hospitals in Anhui; and almost half of hospitals had an efficiency score lower than 0.5 in Guangxi [22].

Although scholars have conducted empirical studies to investigate the efficiency of rural TCM hospitals, some deficiencies still remained: current studies focused on single province using cross-sectional or short panel data [20–27]; majority of these studies applied the traditional DEA model [20–25]. Therefore, this study attempts to investigate the technical efficiency of rural TCM hospitals across China from 2013 to 2018. In detail, we applied super-SBM DEA model to assess the technical efficiency of sample hospitals, then temporal and spatial disparities were further analyzed; besides, the inefficiency existing in inputs and outputs was calculated, and the shadow price of variables were measured.

## Methodology

### Super slacks-based measure DEA model

The DEA model, firstly proposed by Charnes et al., is widely used in gauging technical efficiency (TE) of entity in different fields [28], for the advantages of requiring no specific production function and dealing with multiple inputs and outputs. The basic concept of DEA is to construct a best practice frontier of efficient decision-making units (DMUs: rural TCM hospital in this study) that envelops all inefficient DMUs [29]. Full technical efficiency is achieved by any DMU if and only if none of its inputs can be reduced without worsening its outputs, or none of its outputs can be increased without investing its inputs [30]. In other words, TE obtained by DEA can be interpreted as the ratio of actual to technically maximal output for given inputs, or the ratio of technically minimal to actual inputs for given outputs among DMUs [31]. However, the traditional DEA model only includes the proportional increase (reduction) in outputs (inputs) ignoring slacks in outputs/inputs, which possibly overestimates the efficiency score [29]. Tone [14] proposed SBM DEA model which calculates both proportional changes and slacks of inputs and outputs. The SBM model is specified as follows:

$$\rho =min\frac{1-(\frac{1}{m})\sum_{i=1}^{m}s_{i}^{-}/x_{i0}}{1+(\frac{1}{s})\sum_{r=1}^{s}s_{r}^{+}/y_{r0}}$$ (1)

subject to

$$x_{0}=X\lambda +s^{-}$$

$$y_{0}=Y\lambda -s^{+}$$

$$\lambda \geq 0,\;s^{-}\geq 0,\;s^{+}\geq 0$$

Where X and Y are the input and output matrices, X = (x_ij_)∈R^m*n^, Y = (y_ij_)∈R^s*n^, X>0, Y>0; λ is a nonnegative vector in R^n^, s^-^ and s^+^ indicate input excess and output shortfall which are called slacks; and ρ represents the efficiency of evaluated DMU_0_ (x_0_, y_0_) ranging from 0 to 1. Score closer to 1 implies higher efficiency, and DMUs with score equal to 1 are relatively technical efficient.

The dual program of the original linear program can better illustrate efficiency evaluation with economic interpretation. The dual program to program (1) is:

$$Max\;(uy_{0}-vx_{0})$$ (2)

subject to

uY−vX≤0

$$v\geq \frac{1}{m}*\frac{1}{x_{0}}$$

$$u\geq \frac{1-vx_{0}+uy_{0}}{s}*\frac{1}{y_{0}}$$

The variables u, v are the shadow prices on the constraints in (1), which are also called multipliers or weights in research. “For inefficient units, the shadow price on the output constraint is then directly interpreted as the increase in the efficiency score of a marginal increase in the output variable” [32]. Similarly, the shadow price on the input can be interpreted as the marginal contribution to efficiency improvement.

Tone [15] later came up with super-SBM DEA model to further rank the efficient DMUs. The super-SBM DEA model is applicable to SBM efficient DMUs (ρ* = 1). This model is illustrated as follows:

$$\theta =\min\frac{(\frac{1}{m})\sum_{i=1}^{m}{\bar{x}}_{i}/x_{io}}{(\frac{1}{s})\sum_{r=1}^{s}{\bar{y}}_{r}/y_{r0}}$$ (3)

subject to

$$x_{0}=X\lambda +s^{-}$$

$$y_{0}=Y\lambda -s^{+}$$

$$\bar{x}\geq \sum_{j=1}^{n}\lambda_{j}x_{j},\;\bar{y}\leq \sum_{j=1}^{n}\lambda_{j}y_{j}$$

$$\sum_{j=1}^{n}\lambda_{j}=1$$

$$\bar{x}\geq x_{0},\;\bar{y}\leq y_{0},\;\bar{y}\geq 0,\lambda \geq 0,s^{-}\geq 0,s^{+}\geq 0$$

The connotation of variables (i.e., X, Y, λ) are the same with those in formula (1). In addition, θ is the super-efficiency score obtained by super-SBM DEA model, and similarly higher value represents better performance in efficiency. Therefore, super-SBM DEA model was adopted in this study to capture a more precise and reliable efficiency score through considering slacks of inputs/outputs and discriminating efficient DMUs.

### Indicator selection

Input/output variables selection concerns efficiency evaluation results, and there is no consensus for this selection process. There are three types of frequently chosen input variables, including labor, material and capital resources [33]. However, some research indicated that capital resources had better not be included as inputs in order to distinguish from allocative efficiency [34]. Based on available data and previous literatures [35–38], number of health technicians (NHT), number of non-health technicians (NNHT) and number of actual beds (NAB) were selected as input variables, and number of outpatient/emergency visits (NOEV) and number of inpatient admissions (NIA) as output variables.

The Pearson correlation between inputs and outputs is listed in Table 1. There are significant relations between input and output indicators with correlation coefficient ranging from 0.518 to 0.918 (P<0.01). A positive correlation between input and output variables under 1% significant level is regarded as appropriate for DEA analysis [39]. Therefore, the five selected variables satisfy the DEA requirement.

**Table 1:** Correlation analysis of input and output variables.

Var	NHT	NNHT	NAB
NOEV	0.710***	0.518***	0.649***
NIA	0855***	0.643***	0.918***

Note:

*** P<0.01.

### Study area and data processing

**Fig 1:**
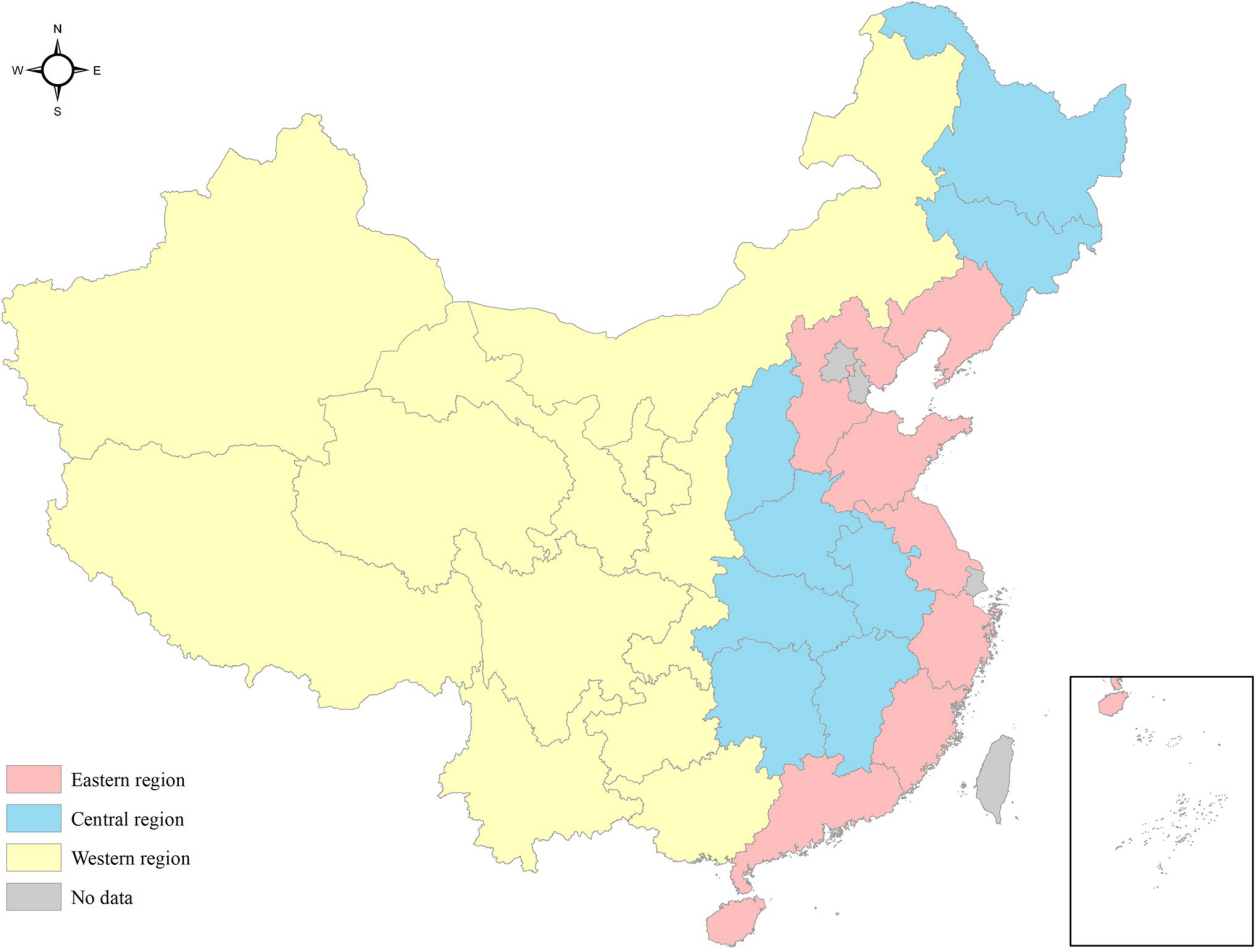
Study sites in this research. This figure is drawn based on the standard map from Resource and Environment Science and Data Center (https://www.resdc.cn/Default.aspx).

This study covered 28 provinces in Chinese mainland. The research site was divided into three regions, the eastern, the central and the western regions, as shown in Fig 1.

Hospital-level data was retrieved from China Statistical Yearbook of Chinese Medicine. Hospitals containing input or output with the value of 0 were dropped from the analysis. To ensure comparability, balanced panel data was built up through removing hospitals not-ever existing during 2013 to 2018. Eventually, 1219 rural TCM hospitals per year was included for efficiency evaluation, with 280 hospitals located in the eastern, 518 in the western and 421 in the central region. Data was processed in MaxDEA Ultra 8.0 software.

## Results

### The temporal and spatial variations of hospital efficiency

#### Time variation

**Fig 2:**
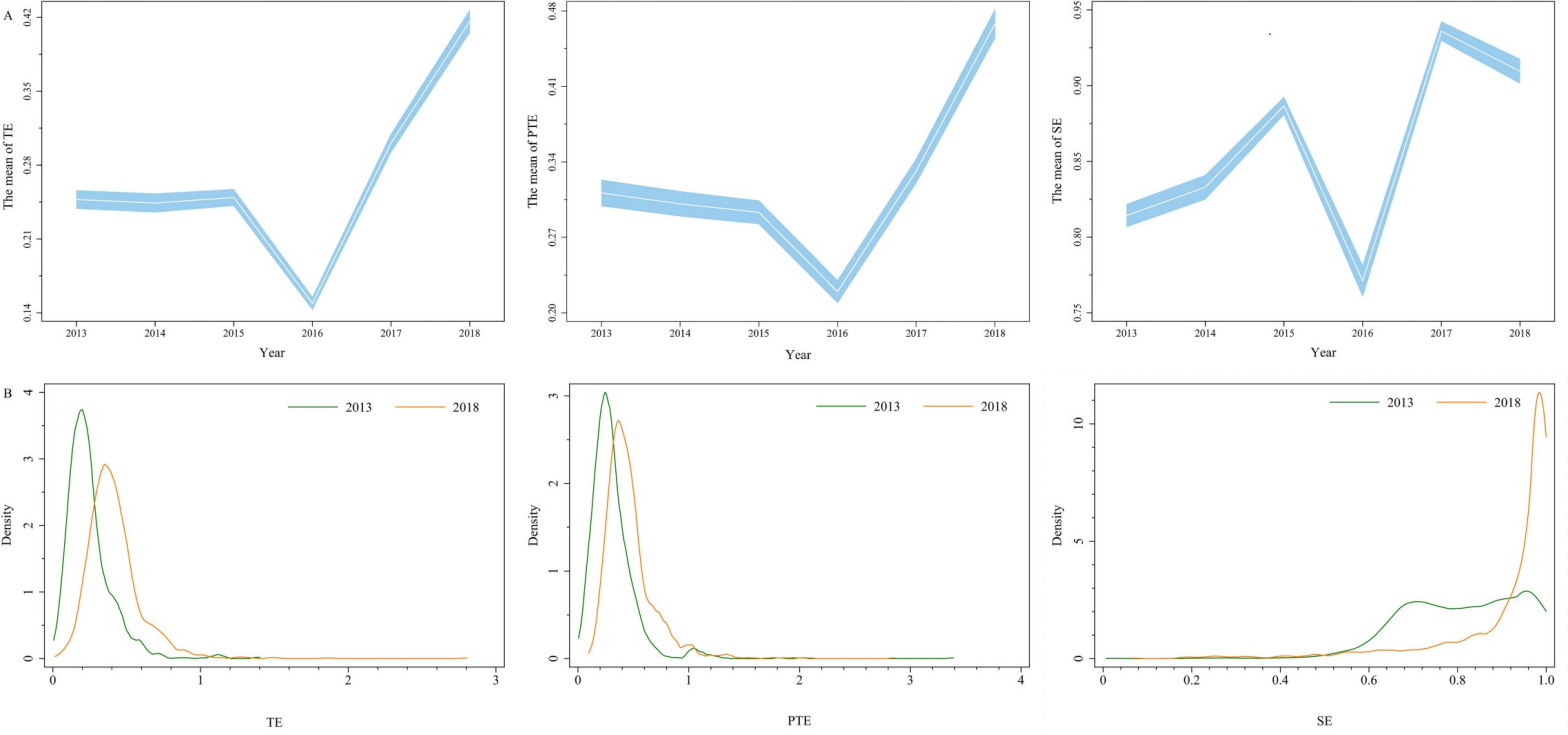
The mean value and kernel density of rural TCM hospital efficiency from 2013 to 2018. A: The mean value of TE, PTE and SE; B: Kernel density curve of TE, PTE and SE.

The average TE of rural TCM hospitals scored at 0.2678. From 2013 to 2015, TE score exhibited a slightly upward trend with fluctuations, followed by an evident decline in 2016 from 0.2493 to 0.1489. Following that, TE score returned to an upward trend and reached a peak of 0.4163 in 2018. The average pure technical efficiency (PTE) score was 0.3208, while scale efficiency (SE) was much higher with a mean value of 0.8584 (Fig 2).

As displayed in Fig 2, both TE and PTE Kernel density curves in 2013 and 2018 were leptokurtic, a large proportion of hospitals scattering between 0.2 to 0.4. Contrarily, SE score of most hospitals dispersed above 0.8. For the return to scales, only up to one-tenth of hospitals operated at an optimal scale. Majority of hospitals experienced decreasing returns to scale except for 2018 (Table 2).

**Table 2:** Number of hospitals in different scale revenue types.

Year	Constant	Decreasing	Increasing
2013	44	1028	147
2014	79	939	201
2015	129	986	104
2016	132	983	104
2017	23	800	396
2018	0	523	696

#### Spatial variation

**Fig 3:**
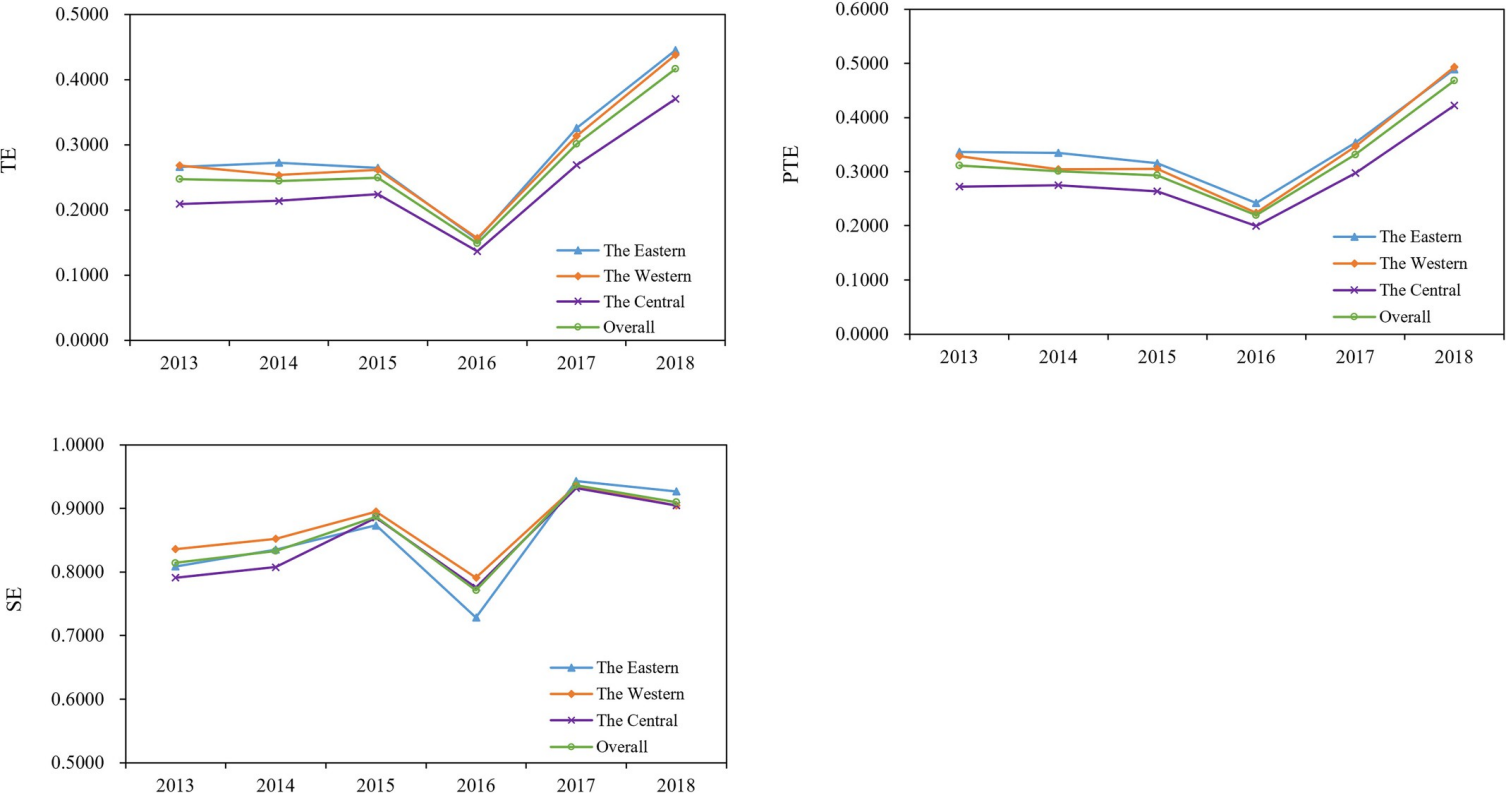
Hospital efficiency in the Eastern, Western and Central regions from 2013 to 2018.

Regional disparity in sample hospital efficiency was shown in Fig 3. For TE, the Eastern (0.2880) had the highest TE score, following by Western (0.2819) and Central (0.2370) regions. It was observed that differences between Eastern-Central and Western-Central gradually reduced from 2013 to 2016 but begun enlarging after 2016. Similarly, the highest PTE score occurred in the Eastern (0.3453), followed by the Western (0.3338) and the Central region (0.2884). As for SE score, the Western region performed the best with a value of 0.8689, following by the Eastern (0.8524) and the Central (0.8494).

**Fig 4:**
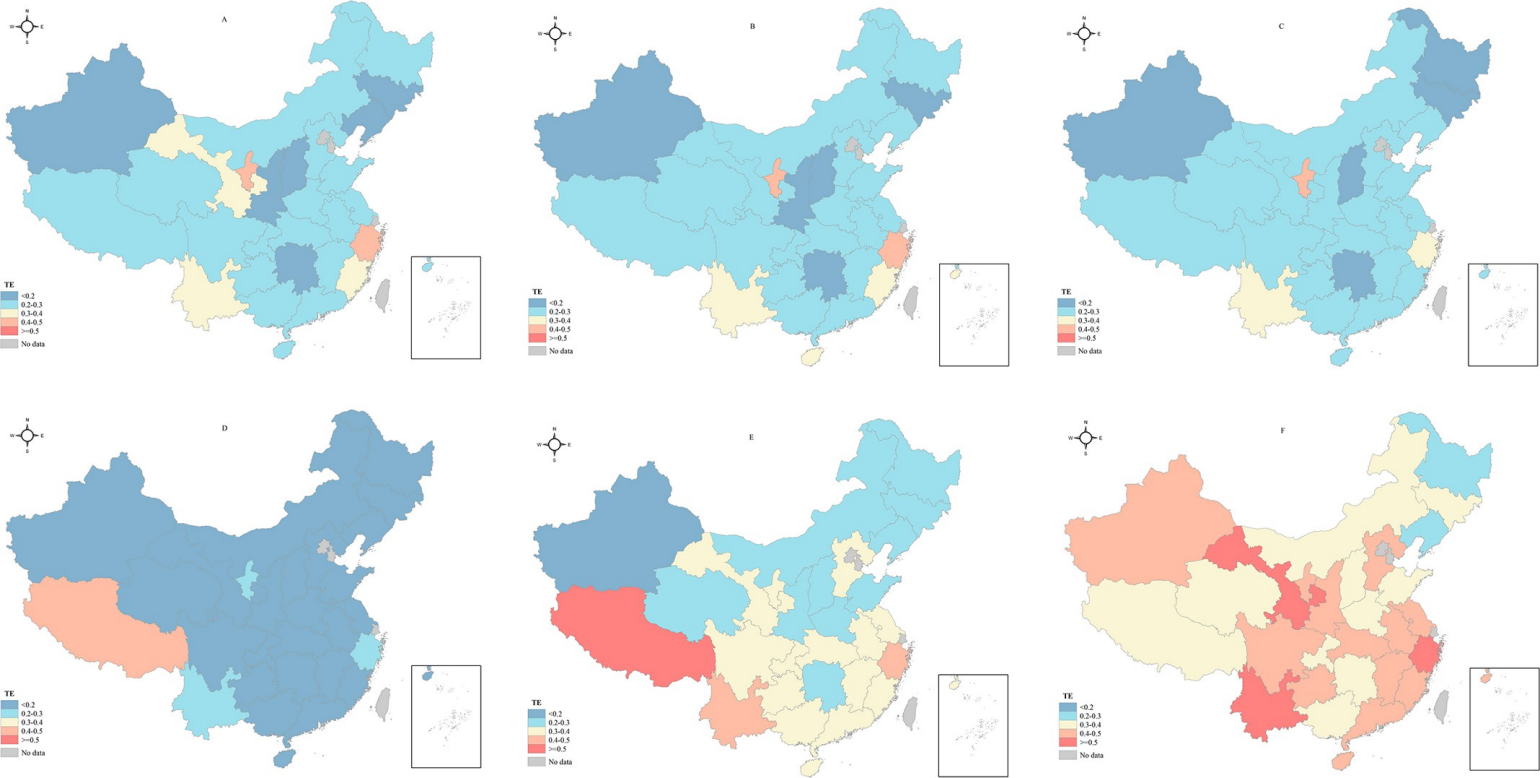
Distribution map of TE in mainland China from 2013 to 2018. A: 2013; B: 2014; C:2015; D:2016; E:2017; F:2018. These figures are drawn based on the standard map from Resource and Environment Science and Data Center (https://www.resdc.cn/Default.aspx).

Additionally, GIS10.7 software was employed to draw provincial distribution maps of TE from 2013 to 2018 (Fig 4). From 2013 to 2015, all 28 provinces obtained TE scores lower than 0.5. Notably, provinces scoring less than 0.2 were mainly concentrated in Hunan, Shanxi, and Jilin from the Central and Xinjiang from the West, while Zhejiang and Ningxia provinces ranked the top with a score close to 0.5. In 2016, TE declined significantly in all provinces, except for Tibet, which scored 0.4296. Since 2017, hospitals in all 28 provinces experienced enhancement in efficiency, especially in 2018. Even, there were three provinces with TE score over 0.5 in 2018, including Yunnan (0.5460), Gansu (0.5596), and Zhejiang (0.7100).

Global and local Moran indices were conducted in GeoDa using Queen contiguity matrix to explore spatial correlation of hospital efficiency. As indicated in Table 3, the value of Moran’s I index slightly increased from 0.1790 in 2013 to 0.1991 in 2018, demonstrating that the positive spatial agglomeration strengthen. However, no spatial correlation was evident from 2014 to 2017.

**Table 3:** Global Moran index of TE from 2013 to 2018.

Year	Moran’s I	Z	P
2013	0.1790	1.8550	0.0440
2014	0.1238	1.4294	0.0820
2015	0.1104	1.3204	0.0980
2016	0.0380	0.7807	0.2120
2017	0.0923	1.0953	0.1450
2018	0.1991	2.0610	0.0350

**Fig 5:**
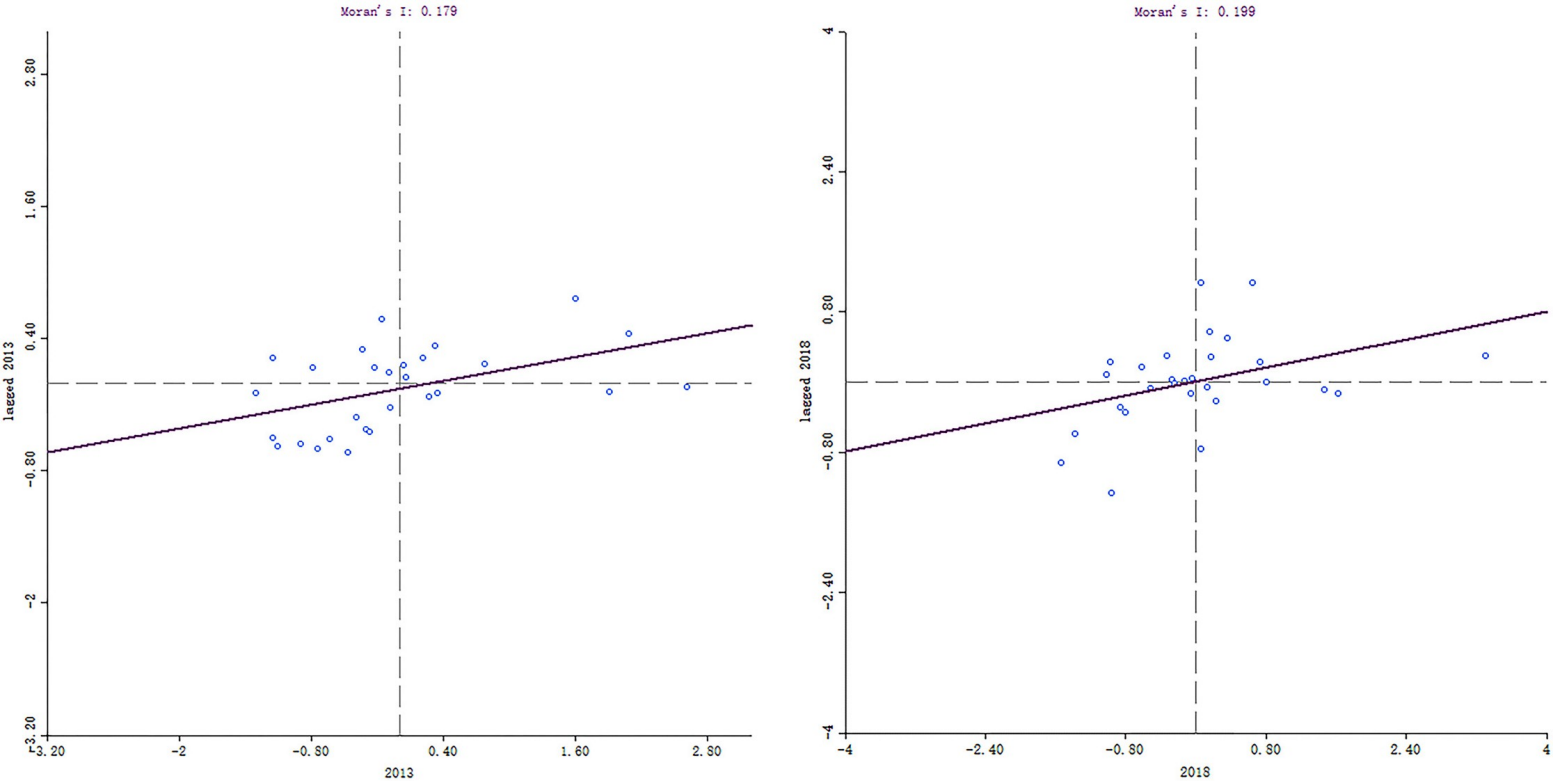
Local Moran scatter diagram of TE in 2013 and 2018.

In the local Moran’s scatter diagram, the first quadrant was H-H spatial correlation pattern, indicating that a high-efficiency region was adjacent to another high-efficiency region, the second quadrant was an L-H pattern, the third quadrant was L-L pattern, and the fourth was H-L pattern. H-H pattern provinces included Zhejiang, Fujian, Sichuan, Anhui, and Jiangxi; L-H pattern provinces were mainly located in the West, including Guangxi, Tibet, Shaanxi, and Xinjiang; L-L pattern provinces included Liaoning, Shandong, Inner Mongolia, Shanxi, Jilin, Heilongjiang, and Henan; and H-L pattern provinces included Hainan and Yunnan (Fig 5).

### Inefficiency of input and output variables

Except for efficiency score, DEA model can measure potential reduction for inputs and improvement for outputs, as well as their projection values. As indicated in Table 4, NNHT had the largest redundancy rate of 60.26%. In addition, redundancy rate of NHT significantly increased from 12.66% to 48.62%, while that of NAB significantly increased from 4.42% to 43.19%. The potential expansion rates for NOEV and NIV were evident, which were 195.40% and 111.82%, respectively. The possible improvement rate of NOEV significantly declined from 442.74% in 2013 to 37.79% in 2018, while that of NIA slightly increased.

**Table 4:** Achievable reductions for inputs and improvement for outputs from 2013 to 2018.

Year	Inputs			Outputs	
	NHT	NNHT	NAB	NOEV	NIA
2013	20.76 (12.66)	15.17 (49.80)	7.97 (4.42)	406501.69 (442.74)	883.10 (14.31)
2014	63.66 (35.93)	16.54 (50.22)	10.16 (5.18)	381454.89 (391.97)	899.84 (13.42)
2015	71.03 (37.29)	22.45 (65.14)	12.24 (5.82)	169897.55 (168.67)	6828.41 (97.94)
2016	113.14 (55.45)	31.31 (84.88)	7.58 (3.41)	55049.18 (52.10)	32790.37 (437.13)
2017	63.75 (29.09)	22.86 (59.28)	28.26 (11.62)	162908.47 (145.93)	4752.03 (57.48)
2018	113.52 (48.62)	20.39 (50.59)	110.66 (43.19)	44314.01 (37.79)	3613.74 (40.65)
Overall	74.31 (37.52)	21.45 (60.26)	29.48 (13.52)	203354.30 (195.40)	8294.58 (111.82)

From regional perspectives, the Eastern region had the highest redundancy rate in personnel, while the Central region had the highest possible reduction rate of NAB. The regional disparity in NOEV was the most evident one, with a potential reduction rate of 262.28% in the Central, which was much higher than 183.77% in the West and 136.59% in the East (Table 5).

**Table 5:** Achievable reductions for inputs and improvement for outputs by regions.

Regions	Inputs			Outputs	
	NHT	NNHT	NAB	NOEV	NIV
Eastern	89.96 (39.97)	24.88 (61.06)	26.21 (11.83)	189916.85 (136.59)	8867.06 (118.77)
Western	58.27 (36.34)	16.81 (59.73)	26.07 (13.50)	162819.70 (183.77)	7224.26 (108.29)
Central	83.47 (36.91)	24.84 (60.17)	35.77 (14.54)	261736.42 (262.28)	9221.28 (111.16)

### Shadow prices of input and output variables

The shadow prices of inputs were reported as follows, while those of outputs were extremely low-value and wasn’t exhibited. As indicated in Table 6, the shadow price of NNHT was the highest with a value of 0.024785, implying that a marginal increase in NNHT would cause an 0.024785 increase in efficiency score for inefficient units. From 2013 to 2018, the shadow price of NNHT remained relatively high compared with the other two inputs.

**Table 6:** The shadow price of inputs from 2013 to 2018.

Year	NHT	NNHT	NAB
2013	0.004283	0.030445	0.004687
2014	0.003705	0.027240	0.004173
2015	0.003612	0.025385	0.003307
2016	0.003481	0.025620	0.003537
2017	0.003007	0.022322	0.003109
2018	0.002458	0.017701	0.003857
Overall	0.003424	0.024785	0.003778

As shown in Table 7, marginal contribution of NNHT to efficiency improvement was more evident in all regions. Concerning regional disparities, the shadow price of NNHT is significantly higher in the West with a value of 0.033527 comparing with the Central (0.019091) and the East (0.017285).

**Table 7:** The shadow price of inputs by regions.

Year	NHT	NNHT	NAB
The East	0.002606	0.017285	0.003396
The West	0.004426	0.033527	0.004473
The Central	0.002745	0.019091	0.003182

### Robustness test

**Fig 6:**
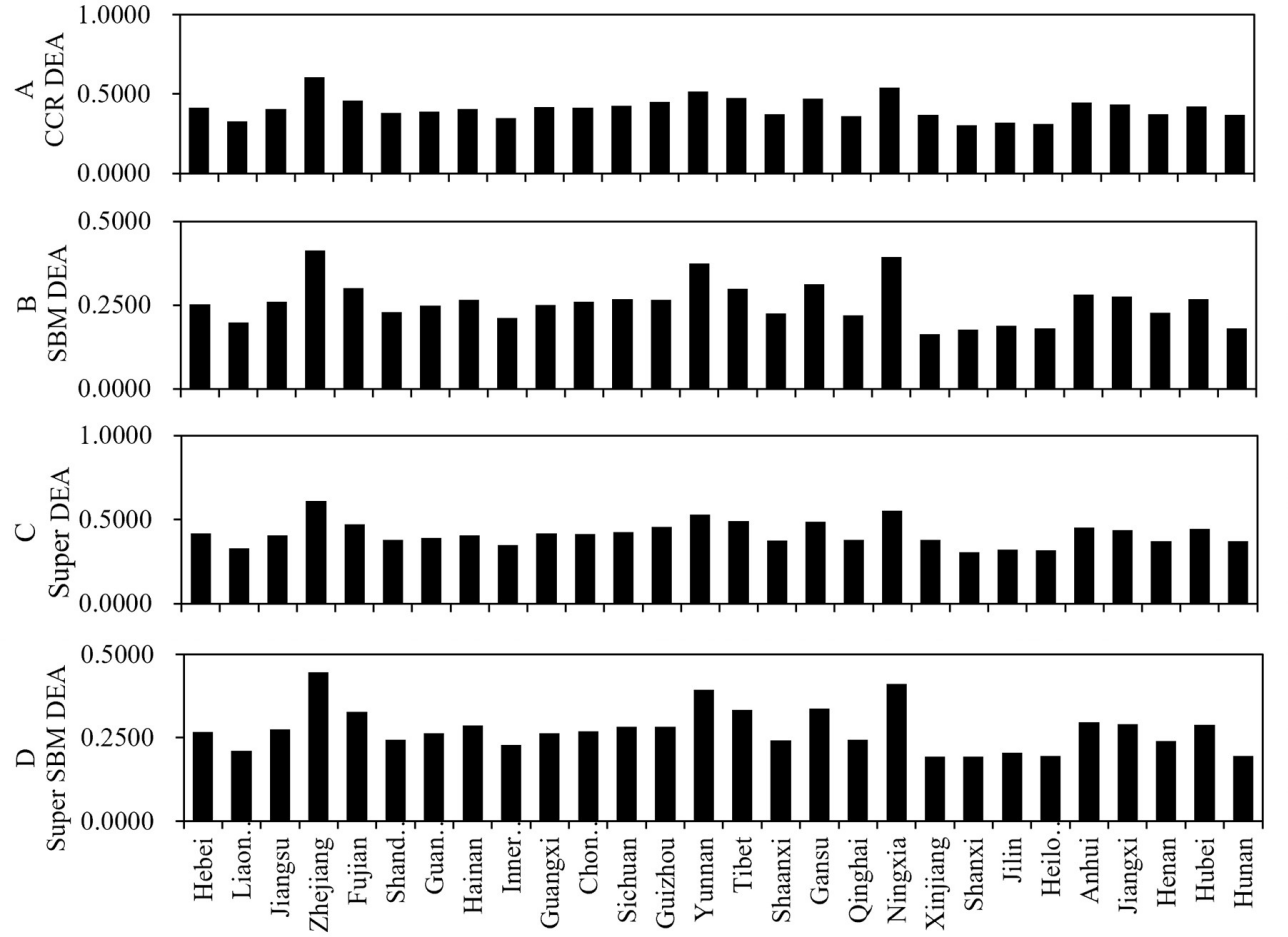
Provincial mean value of hospital efficiency of different DEA models by CCR, SBM, super and super SBM DEA model.

To verify the robustness of our findings, we measured sample hospitals’ efficiency using three representative DEA methods, including CCR, SBM, and super DEA models. As illustrated in Fig 6, the efficiency variations among 28 provinces calculated by CCR, SBM, and super DEA models were similar to our findings. Therefore, the results of this research were reliable and robust.

## Discussion

A nationwide efficiency evaluation on rural TCM hospitals from 2013 to 2018 was implemented using super-SBM DEA model, yielding some meaningful and insightful findings. Rural TCM hospitals exhibited an average efficiency value of 0.2678 and even a large proportion of hospitals dispersed between 0.2 and 0.4, implying serious waste of health resources and insufficient health care provision of sample hospitals. There is ample room of efficiency improvement for rural TCM hospitals, which has already been confirmed in prior studies [20–23, 26]. The reasons behind low efficiency of rural TCM hospitals are complicated and multifaceted. It has been found that financial burden is one influential factor for ineffectiveness in hospitals [40]. Revenue of rural TCM hospitals is significantly less than that of rural general hospitals [41]. Zero mark-up drug policy carried out in 2012 is a huge attack to hospital revenue, especially for TCM hospitals whose drug revenue accounts for a large proportion in total amount [42]. This measurement might aggravate the financial burden of rural TCM hospitals unless corresponding reimbursement system being well-designed and implemented. There are also problems rooted within hospitals. As shown, average PTE score of sample hospitals was merely 0.3208 and much lower than SE score (0.8584), indicating low PTE is the major obstacle for inefficiency of rural TCM hospitals [21, 26, 43]. PTE generally relates with two factors: one is medical technology, and the other is management level within hospitals [29]. Rural areas have difficulties in attracting health professionals because of lower salary and worse career prospect. Among rural TCM hospitals, only 3% of health technicians obtained master or doctoral degrees, and 17% obtained bachelor’s degrees [44]. Besides, without adequate financial support, rural hospitals are unable to purchase advanced medical equipment [42]. Lack of health professionals and advanced technology impedes service capacity of these hospitals. On the other hand, there is a dearth of professional managers and well-designed administrative system in rural TCM hospitals [45]. The current administrative staff is generally transferred from clinical departments, and they are not familiar with the operation and management of hospitals [46]. Therefore, PTE is of great significance in eliminating inefficiency of rural TCM hospitals. The issue of over-expansion in rural TCM hospitals also deserves much attention [47], with a large of proportion of sample hospitals operating with decreasing returns to scale. Hospital size should take full considerations of population density, geographical location, and other factors [48].

Redundancy and insufficiency co-exist in input and output variables among inefficient hospitals, respectively. The potential reductions for NHT, NNHT, and NAB were 74.31 (37.52%), 21.45 (60.26%), and 29.48 (13.52%), respectively. The personnel redundancy was more serious in rural TCM hospitals from an input standpoint, which was in accordance with past literature [20]. Optimizing inner management system is found to be a feasible and effective measure to alleviate health resource waste [49]. On the view of outputs, insufficient volume in outpatient service is more prominent. In economically underdeveloped rural areas, patients might be reluctant to receive treatment unless hospitalization is inevitable, resulting in low utilization of outpatient service [50]. Besides, shadow price, which implies the marginal contribution of input/output towards efficiency improvement, is not frequently explicitly exhibited in papers on DEA [51], particularly in hospital efficiency evaluation. The shadow price of NNHT was relatively higher with an average value of 0.024785, implying a reduction in NNHT by one unit leading to the improvement of 0.024785 in efficiency score.

Although the overall efficiency of rural TCM hospitals was unsatisfying, it turned out to make some progress in the long term. An overall upward trend was identified from 0.2474 in 2013 to 0.4163 in 2018. The improvement in rural TCM hospitals’ performance has also been confirmed in other literatures. For example, an obvious growth in hospital efficiency was identified from lower than 0.5 to 0.686 during 2001–2017 in Hubei province [52], and efficiency improvements were also observed in Henan [26] and Shandong province [25]. The increase might benefit from the new round of rural hospital reform implemented in 2012 [53], which highlights the role and increases investment for rural TCM hospitals. It was also noted that an evident decline in efficiency appeared in 2016. Here are the possible explanations for this phenomenon. Government subsidy acts as an impetus for hospital efficiency [54]. As reported, the growth rate in financial subsidy for TCM hospitals slightly slowed down in 2016 (19.64%) comparing with that in 2015(28.47%) and 2014(22.52%), which might lead to hospital efficiency decrease as a result. In addition, government integrated urban-rural medical insurance to eliminate its reimbursement gap at the beginning of 2016. While this might cause rural residents flow into higher-level hospitals at initial stage of policy implementation [49]. Even though, rural TCM hospitals’ efficiency shows a favorable sign of progress in an overall view.

Finally, rural TCM hospitals efficiency displayed provincial and regional disparities. A positive spatial aggregation was observed in hospital efficiency; in other words, high-efficiency provinces assemble, and low-efficiency provinces assemble. It was reported previously, the regional coordinated development strategy in 2014 eased the unbalance in rural health system in short period [55]. This disparity is still noticeable, as the global Moran’s index grew to 0.1991 in 2018. High-high efficiency cluster generally dispersed in eastern coastal provinces (including Zhejiang and Fujian) and their neighboring provinces such as Anhui and Jiangxi. Spatial spillover effect has been confirmed in health system efficiency [56]. Traffic accessibility is found to strengthen regional interdependence of health resources [57], therefore, eastern coastal provinces with high efficiency might exert positive impact on their neighbors. And low-low efficiency cluster mainly located in following central region Inner Mongolia, Shanxi, and Heilongjiang, which was consistent with one similar research [56]. From the perspective of region variation, the eastern region performed the best in TE and PTE, whereas the central region exhibited the lowest TE, PTE, and SE scores. Additionally, it was observed that western region ranked the top in SE. Prior studies also found similar results [58, 59]. This heterogeneity is determined by some socio-economic factors [60]. According to census statistics, the eastern region is the most densely populated area, and the western region is the most sparsely area in contrast. Population density was found to be positively correlated with TE [61], but an obstacle role for SE [62] among hospitals. A larger population triggers more medical demands, which not only promotes health resource utilization, but also induce the risk of over expansion in hospital scale. Besides, local economic level is a substantial influential factor for hospital efficiency [63, 64]. The eastern region, as the most prosperous area in China, is able to introduce advanced equipment and attract excellent personnel, which are the key drivers for TE. These factors result in regional difference for hospital efficiency, and government should vigorously carry out effective strategy to mediate the unbalanced phenomenon.

## Conclusion

The super-SBM DEA model was employed to assess efficiency of rural TCM hospitals in China from 2013 to 2018. Although TE of sample hospitals presented an increasing trend, the performance of rural TCM hospitals was worrying with an average TE score of merely 0.2678. There existed wastes in inputs, especially in personnel variables, as well as deficiency in outputs. Besides, it was found that optimizing inputs brought in a more substantial marginal improvement in hospital efficiency, which should be considered by policymakers. Finally, spatial disparities existed in rural TCM hospital efficiency across provinces and regions, which deserves much attention for the balanced development in hospital efficiency.

## Abbreviations

DEA: data envelopment analysis
SBM: slack-based measure
TCM: traditional Chinese medicine
TE: technical efficiency
PTE: pure technical efficiency
SE: scale efficiency
DMUs: decision-making units
NHT: number of health technicians
NNHT: number of non-health technicians
NAB: number of actual beds
NOEV: number of outpatient/emergency visits
NIA: number of inpatient admissions
